# Maged1, a new regulator of skeletal myogenic differentiation and muscle regeneration

**DOI:** 10.1186/1471-2121-11-57

**Published:** 2010-07-20

**Authors:** Tuan HN Nguyen, Mathieu JM Bertrand, Christiane Sterpin, Younes Achouri, Olivier RY De Backer

**Affiliations:** 1 URPHYM (Unité de Recherche en Physiologie Moléculaire), NARILIS (Namur Research Institute for Life Sciences), FUNDP school of Medicine, University of Namur, 21 rue de Bruxelles, Namur B-5000, Belgium; 2 Molecular Signaling and Cell Death Unit, Department for Molecular Biomedical Research, VIB, 9052 Ghent, Belgium; 3 Department of Biomedical Molecular Biology, Ghent University, 9052 Ghent, Belgium; 4 Christian de Duve Institute of Cellular Pathology, Université Catholique de Louvain (UCL), Avenue Hippocrate 75, Brussels B-1200, Belgium

## Abstract

**Background:**

In normal adult skeletal muscle, cell turnover is very slow. However, after an acute lesion or in chronic pathological conditions, such as primary myopathies, muscle stem cells, called satellite cells, are induced to proliferate, then withdraw definitively from the cell cycle and fuse to reconstitute functional myofibers.

**Results:**

We show that Maged1 is expressed at very low levels in normal adult muscle but is strongly induced after injury, during the early phase of myoblast differentiation. By comparing in vitro differentiation of myoblasts derived from wild-type or Maged1 knockout mice, we observed that Maged1 deficiency results in reduced levels of p21^CIP1/WAF1^, defective cell cycle exit and impaired myotube maturation. In vivo, this defect results in delayed regeneration of injured muscle.

**Conclusions:**

These data demonstrate for the first time that Maged1 is an important factor required for proper skeletal myoblast differentiation and muscle healing.

## Background

Skeletal muscle contains quiescent mononucleated myogenic cells, called satellite cells, that are able to proliferate in response to injury and give rise to new functional myofibers. Soon after a lesion, inflammatory cells such as neutrophiles and macrophages are recruited at the damaged site where they release growth factors and cytokines that induce satellite cells to proliferate. The activated satellite cells divide asymmetrically to (1) reconstitute the stock of stem cells and (2) provide myoblasts that will first proliferate and then undergo myogenic differentiation and maturation to form new muscle fibers. The new cells can fuse between themselves or with existing fibers. Muscle differentiation involves a cascade of muscle-specific gene activation coordinated with irreversible withdrawal from the cell cycle. The commitment of cells to the muscle lineage and to progression through differentiation requires the activation of a limited set of muscle-specific transcription factors. These key factors are the basic helix-loop-helix (bHLH) transcription factors MyoD, Myf5, myogenin (MyoG) and MRF4, which are collectively called myogenic regulatory factors (MRFs). The MRFs activate their target genes by binding to the E-box CANNTG consensus sequence [1-4]. Primary cultures of newborn or adult skeletal muscles or established muscle cell lines like C2C12 can be used to study myogenic differentiation. These cells can be maintained as dividing cells in serum rich medium (GM), but under the condition of mitogen deprivation, they exit the cell cycle in G1 to G0, express the repertoire of muscle-specific genes and fuse to form syncytial myotubes [5,6]. MyoD is expressed early after satellite cell activation. In dividing cells, its function is repressed, but upon induction of differentiation, its transcriptional activity is induced, and it governs two distinct essential functions: (1) activation of muscle specific genes, such as MyoG, and (2) induction of withdrawal from the cell cycle by activation of the cell cycle inhibitors p21^CIP1/WAF1 ^and Rb [7,8].

Necdin, a member of the large family of Mage proteins, has recently been reported to promote myoblast survival and differentiation and to improve skeletal muscle regeneration [9,10]. Necdin has also been shown to protect muscle fibers from tumor-induced wasting by interfering with the TNF signaling cascade [11]. Maged1 (also called Nrage and Dlxin1) is an X-linked Mage family member that physically interacts with Necdin [12]. Although in vitro studies have suggested a role for Maged1 in diverse biological functions, only its role in the apoptotic signaling cascade emanating for the TNF receptor superfamily member p75NTR (p75 NeuroTrophin Receptor) has been confirmed in vivo so far [13-15]. Interestingly, data obtained by high throughput expression analysis indicate that the expression of *MAGED1 *RNA is upregulated in the muscles of Duchenne muscular dystrophy (DMD) patients, a condition in which muscle regeneration concomitantly occurs with the degeneration of myofibers [16]. This observation and the fact that Maged1 physically interacts with Necdin encouraged us to investigate the possible roles of Maged1 in muscle regeneration and differentiation. Here, we show that Maged1 is transcriptionally upregulated during myogenic differentiation and that Maged1 deficiency results in deficient myoblast differentiation and maturation, leading to delayed muscle regeneration in vivo.

## Results

### Maged1 is induced early during myogenic differentiation

**Figure 1:**
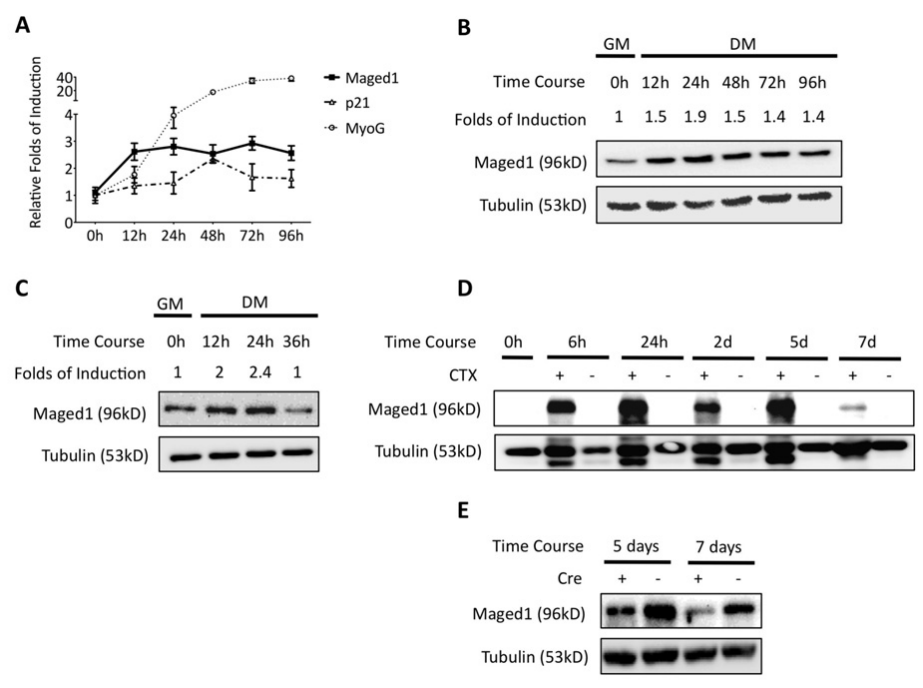
**Maged1 is induced early during myogenic differentiation**. (A) *Maged1*, *p21^CIP1/WAF1 ^*and *MyoG *RNA levels were determined using qRT-PCR during the differentiation of C2C12 myoblasts induced by serum starvation. RNA levels were normalized using *GAPDH *RNA as the reference. Data are presented as the relative folds of induction at different time points with respect to the level measured at the time of serum starvation (0 h). (B-E) Immunoblotting analysis of Maged1 in C2C12 myoblasts (B) and primary mouse myoblasts (C) during myogenic differentiation and in TA muscle regenerating after CTX injection in wild-type mice (D) and in *Maged1^tm1Urfm/HSA-Cre ^*mice that have a cell-type specific deletion of Maged1 in myoblasts (E). Induction of Maged1 was quantified by digital imaging using alpha-tubulin for normalization (B and C).

Microarray gene profiling indicated that Maged1 was upregulated in skeletal biopsies from DMD patients when compared to unaffected controls [16]. To determine if Maged1 was expressed in proliferating and/or differentiating muscle cells, we first analyzed Maged1 RNA and protein levels in myoblasts from the C2C12 cell line. Differentiation of the myoblasts was induced by serum starvation and monitored by following expression of *p21^CIP1/WAF1 ^*and *MyoG *during a four-day period. We observed that Maged1 was expressed in proliferating C2C12 cells and that Maged1 RNA and protein levels increased, respectively, up to three and two folds after induction of differentiation. The levels of Maged1 reached a plateau 24 hours after serum starvation, at the time when the cells withdraw from the cell cycle, and these elevated levels were maintained until the end of the experiment, four days after serum starvation (Figure 1A-B and Additional file 1: Figure S1). We next analyzed expression of Maged1 in primary myoblast cultures derived from mouse tibialis anterior (TA) muscle and found Maged1 protein expressed in the proliferating cells. A two-fold induction of Maged1 expression was also observed in these cells following differentiation by serum starvation (Figure 1C). To determine if Maged1 is induced in vivo in regenerating muscle, we followed its expression during the course of muscle healing after injection of cardiotoxin (CTX) in TA muscles. Western blot analysis detected no or very little Maged1 expression in intact mouse muscle, but the protein was strongly induced as soon as 6 hours after CTX injection (Figure 1D). This Maged1 upregulation could emerge from muscle cells (mature myofibers and/or activated satellite cells) or from non-muscle cells (e.g. inflammatory cells or endothelial cells). To determine the contribution of muscle cells to the observed induction, we followed Maged1 expression after CTX injection in TA muscle of mice with a Cre-mediated deletion of Maged1 driven by the promoter of the human alpha-skeletal actin (HSA) gene, which is activated specifically in differentiating muscle cells [17]. These *Maged1^tm1Urfm/HSA-Cre ^*mice were obtained by crossing mice with a conditional (floxed) allele of Maged1 (*Maged1^tm1Urfm/tm1Urfm^*) with transgenic mice carrying an HSA-Cre transgene (Tg(ACTA1-cre)79Jme/J). In the resulting mice, Cre is switched on and Maged1 is deleted specifically in differentiating muscle myotubes [17]. The weaker induction of Maged1 in these mice demonstrated that differentiating and/or mature muscle cells contribute to the Maged1 induction after CTX injury (Figure 1E). The remaining Maged1 expression most probably originates from muscle cells that do not express Cre (e.g. activated satellite cells and proliferating myoblasts) and/or from non-muscle cells. Together, our results demonstrate in vitro and in vivo induction of Maged1 in the course of myoblast differentiation.

### Maged1 transcription is activated by MRFs during myoblast differentiation

**Figure 2:**
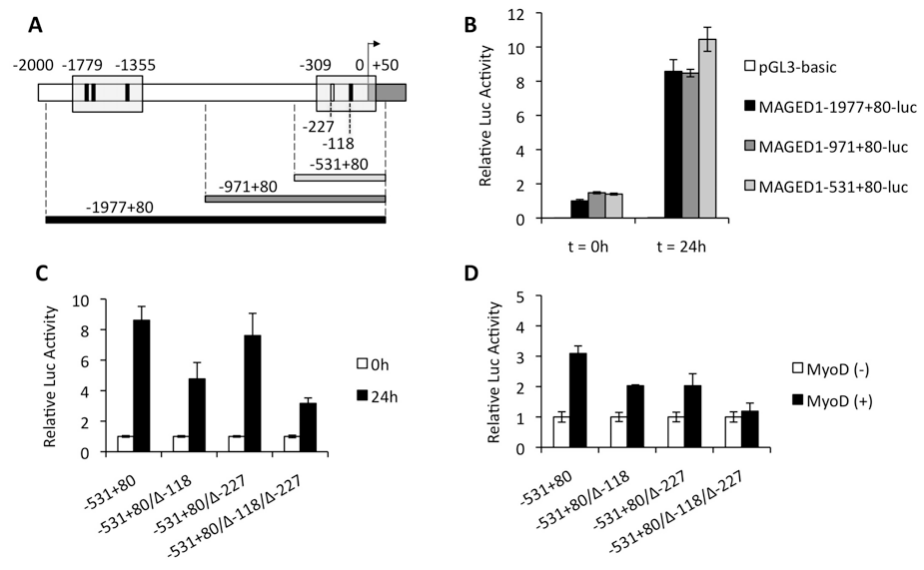
**The *MAGED1 *promoter is activated during myogenic differentiation and contains two E-boxes required for MyoD-dependent transcription**. (A) Three promoter fragments of the human *MAGED1 *gene were amplified using PCR and inserted upstream from the firefly Luc reporter gene into pGL3-basic. Regions of the promoter conserved in mammals are boxed. E-box consensus sequences are indicated by vertical boxes (black if conserved in mammals). Coordinates are shown with respect to the first nucleotide of the *MAGED1 *transcript. (B) The 3 reporter constructs (pMAGED1-1977+80-luc, pMAGED1-971+80-luc, pMAGED1-531+80-luc) were used to transfect C2C12 myoblasts. Twenty-four hours after transfection, cells were shifted to DM and cultivated for another 24 hours before luciferase activity quantification. Firefly luciferase activity was normalized by the *Renilla *luciferase activity specified by a *Renilla *luciferase expression plasmid (pRL-CMV) co-transfected with the MAGED1-luc constructs. Results are presented as relative luciferase activity with respect to the activity of the MAGED1-1977+80-luc construct at the time of serum starvation (t = 0 h). (C) Effect of mutation of the -227 and -118 E-boxes on the luciferase activity of pMAGED1-531+80-luc. Experimental procedure was the same as in B. (D) Effect of MyoD on the promoter activity of the -531+80 fragment with intact or mutated -227 and -118 E-boxes. 3T3 fibroblasts were transfected with the MAGED1-luc constructs with or without the MyoD expression plasmid pEMSV-MyoD. Results are presented as relative luciferase activities with respect to the activity of the promoter fragments in absence of MyoD.

The myogenic differentiation program is regulated primarily by the MRFs that all bind to E-box motifs in their target gene promoters. These factors act in cooperation with myocyte enhancer factor 2 (MEF2) and bHLH E proteins to drive the myogenic differentiation program. In C2C12 myoblasts, MyoD is expressed but is inactive when cells are cultured in serum-rich medium [18,19]. Upon serum starvation, MyoD is activated and triggers the myogenic differentiation program by transactivating its target genes, including *MyoG*. The early upregulation of *Maged1 *RNA after serum starvation suggested that *Maged1 *transcription could be regulated by MyoD. We analyzed the promoter region of the human *MAGED1 *gene (from -1977 to +80) *in silico *and compared it with the orthologous regions of distant species of mammals: *M. musculus, S. scrofa *and *C. familiaris*. We found that the *MAGED1 *promoter of all of these species contains four conserved E-boxes located in two conserved regions, suggesting that these sites could be important for the control of Maged1 transcription (Figure 2A).

To test this hypothesis, the DNA fragment from -1977 to +80 of the human *MAGED1 *promoter was inserted upstream of the firefly luciferase reporter gene and tested for *cis*-activation in proliferating and differentiating C2C12 myoblasts. We found that the luciferase activity controlled by this fragment increased approximately ten fold 24 hours after the induction of differentiation by serum starvation. To localize the key regulatory elements in this fragment, we deleted segments from its 5' end and found that the -531+80 fragment had nearly the same activity as the -1977+80 fragment (Figure 2A-B). This indicates that the two proximal E-boxes at -227 and -118 (especially the box at -118, which is conserved in mammals) are possible important sites for transcriptional induction. We tested the importance of these two E-boxes by targeted mutagenesis. Mutation of the -118 E-box resulted in a significant reduction of induction after serum starvation, indicating that this site is important for induction and that MRFs are probably directly involved (Figure 2C). We then tested the potential of transfected MyoD to transactivate the -531+80 fragment in NIH-3T3 cells, which do not express endogenous MyoD. MyoD induced a three-fold increase in the expression of the luciferase reporter (Figure 2D and Additional file 2: Figure S2). Altogether, these results show that the *MAGED1 *promoter is activated upon the induction of myoblast differentiation and contains conserved E-boxes that are important for its transactivation by MyoD.

### Deficiency of Maged1 results in impaired myogenic differentiation

**Figure 3:**
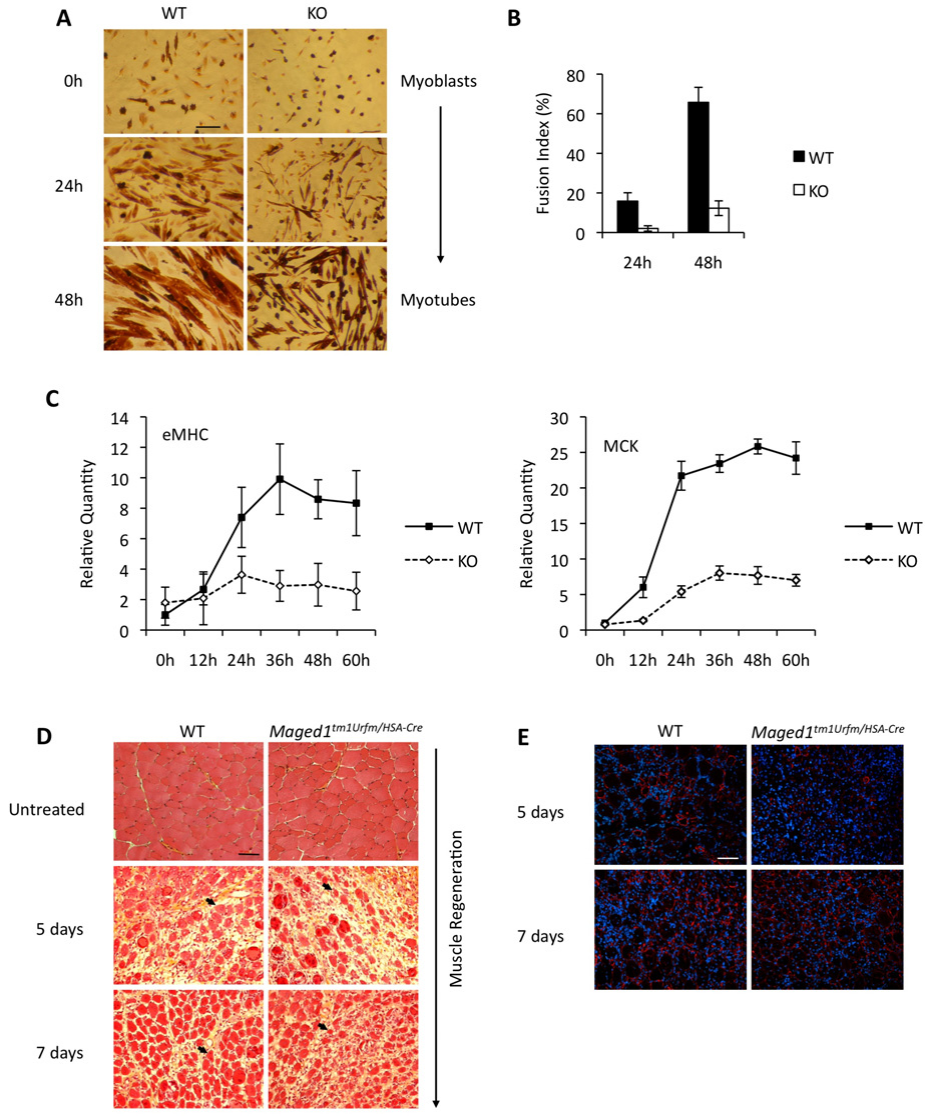
**Deficiency of Maged1 results in impaired myogenic differentiation**. (A) Primary myoblasts derived from wild-type or Maged1 knockout mice were cultivated on collagen-coated plates and induced to differentiate by serum starvation. Cells were stained with anti-MHC antibody. Images were photographed under a light microscope. Scale bar, 100 μm. (B) The fusion index was calculated by dividing the number of nuclei in MHC-positive cells containing at least 2 nuclei by the total number of nuclei in MHC-positive cells. (C) *eMHC *and *MCK *RNA levels during differentiation were analyzed using qRT-PCR. Data are presented as the relative folds of induction with respect to wild-type myoblasts at the time of serum starvation (t = 0 h). *GAPDH *RNA was used for the normalization. (D) Representative sections analyzed by H&E staining of TA muscles of *Maged1^tm1Urfm/HSA-Cre ^*mice after CTX injection. Arrows indicate newly formed myotubes. Scale bar, 50 μm. (E) Laminin expression (red) analyzed by immunostaining of TA muscles of *Maged1^tm1Urfm/HSA-Cre ^*mice after CTX injection. Nuclei were visualized by Hoechst 33342. Scale bar, 50 μm.

The induction of Maged1 during myoblast differentiation and the fact that the related protein Necdin promotes myoblast differentiation suggested that Maged1 could also be a regulator of myogenic differentiation. To investigate this possibility, we compared differentiation of primary myoblasts isolated from wild-type or from constitutive Maged1 knockout mice [14]. We observed that wild-type myoblasts differentiated and matured to form myotubes 48 hours after serum starvation. In contrast, very few and very thin myotubes were formed by Maged1-deficient myoblasts at this time point (Figure 3A). We quantified this deficiency to form myotubes by calculating the fusion index (the fraction of nuclei present in multinucleated myotubes) 24 hours and 48 hours after serum starvation. As shown in Figure 3B, we observed a dramatic reduction of the fusion index at these two time points, confirming that Maged1 is essential for the fusion of myoblasts in these conditions (Figure 3B). Using qRT-PCR, we also followed the expression of embryonic myosin heavy chain (eMHC) and muscle creatine kinase (MCK), two late markers of myoblast differentiation. We observed that the impaired morphological differentiation of the Maged1-deficient myoblasts was accompanied by a largely reduced induction of these two markers (Figure 3C). We then analyzed the regeneration capacity of injured muscle in mice with the HSA-Cre mediated deletion of Maged1 (*Maged1^tm1Urfm/HSA-Cre^*). Although no overt morphological abnormality was observed in the untreated Maged1-deficient muscles, we observed that muscle regeneration was significantly delayed: five days after CTX injection in the TA muscle, fewer and smaller new myofibers had been formed and the laminin scaffold was much more disorganized when compared to controls (Figure 3D-E). We quantified this defect by measuring the cross-sectional area of the new muscle fibers and found that it was reduced by 47% in *Maged1^tm1Urfm/HSA-Cre ^*mice compared to controls (44 ± 7 μm^2 ^vs. 82 ± 9 μm^2^). Two days later (7 days after CTX injection), the difference between wild-type and *Maged1^tm1Urfm/HSA-Cre ^*had become smaller (Figure 3D-E). Altogether, these observations demonstrate that Maged1 is essential for myoblast differentiation in vitro and that Maged1 deficiency delays muscle regeneration in vivo.

### Maged1 is required for normal p21^CIP1/WAF1 ^expression and cell cycle withdrawal of differentiating myoblasts

**Figure 4:**
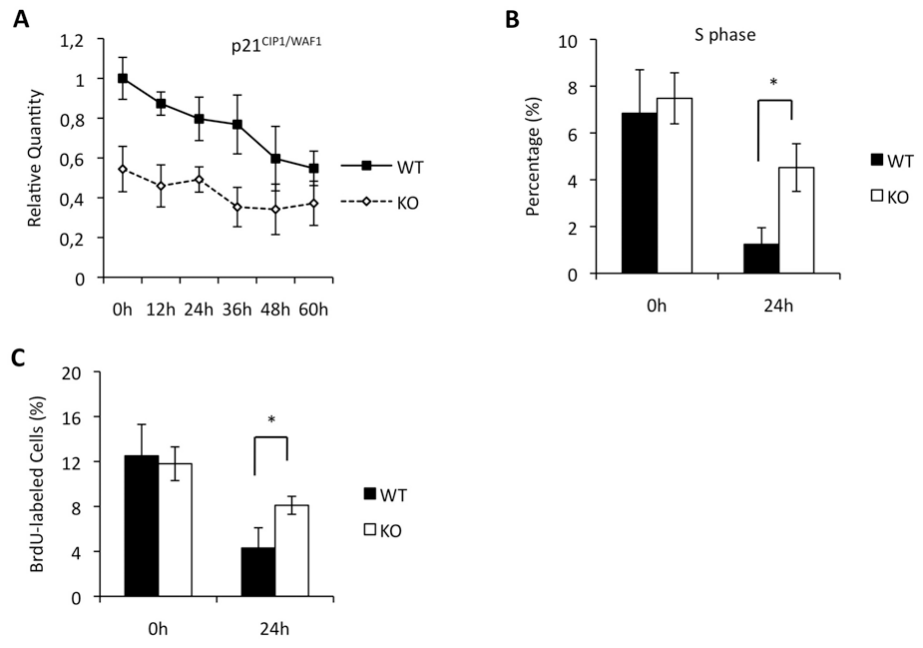
**Maged1 knockout myoblasts have a lower *p21^CIP1/WAF1 ^*expression level and fail to properly exit from the cell cycle**. Wild-type and Maged1 knockout primary myoblasts were induced to differentiate by serum starvation. (A) The *p21^CIP1/WAF1 ^*RNA level was quantified by qRT-PCR. *GAPDH *RNA was used for normalization. Data are presented as the ratio relative to the *p21^CIP1/WAF1 ^*RNA level in wild-type cells at the time of serum starvation (t = 0 h). (B) Fractions of wild-type and Maged1-deficient myoblasts in S phase in GM (0 h) and 24 h after serum starvation. Cells were analyzed by FACS after staining with propidium iodide. (*p < 0.01). (C) BrdU incorporation of differentiating myoblasts was analyzed after 24 h in DM. Data are presented as the fraction of BrdU-labeled cells. (*p < 0.01).

Reduced differentiation could result from deficient expression of MRFs or from an inability to withdraw from the cell cycle in G0. To identify the cause of the differentiation defect observed in the myoblasts derived from constitutive Maged1 knockout mice, we analyzed the expression of *p21^CIP1/WAF1^*, *MyoD*, *MyoG *and *Mef2C *by qRT-PCR. We did not detect significant differences in the levels of *MyoD*, *MyoG *and *Mef2C *between knockouts and controls (Additional file 3: Figure S3). However, we observed a reduced level of *p21^CIP1/WAF1 ^*mRNA in both proliferating and differentiating Maged1 knockout myoblasts (Figure 4A). p21^CIP1/WAF1 ^facilitates cell cycle withdrawal and enhances the survival of differentiating myoblasts [20,21]. We hypothesized that the reduced level of *p21^CIP1/WAF1 ^*in Maged1-deficient myoblasts could impair cell cycle withdrawal. To test this possibility, we compared the cell cycle distribution of wild-type and Maged1-deficient myoblasts upon induction of the differentiating. FACS analysis showed no difference in cell cycle profiles in serum rich medium. However, 24 hours after serum starvation, the fraction of cells in S phase was three times greater in the Maged1-deficient cell population when compared to the wild-type control cells (Figure 4B). BrdU incorporation confirmed that Maged1-deficient myoblasts do not exit the cell cycle efficiently upon serum starvation: twice as many Maged1 deficient cells incorporated BrdU compared to wild-type cells (Figure 4C). We concluded that Maged1 knockout myoblasts cannot exit the cell cycle efficiently after the induction of differentiation.

## Discussion and Conclusions

Skeletal muscle satellite cells are quiescent stem cells that are activated in response to physiological or pathological stimuli such as after muscle injury or in chronic pathological conditions. After an acute lesion, the initial phase of muscle repair is characterized by inflammation and degeneration of the damaged tissue. Almost simultaneously, satellite cells are activated and proliferate to generate a population of myoblasts that differentiate and fuse to form new multinucleated myotubes. Muscle regeneration is a tightly orchestrated process, and understanding the molecular and cellular mechanisms responsible for its control is important for the development of treatments of muscles impaired by aging, disease or atrophy.

### Maged1 is upregulated during myoblast differentiation and muscle regeneration

Maged1 is silent in normal adult muscles, but it is massively induced soon after injury of skeletal muscles. In primary cultures of myoblasts, Maged1 is expressed in proliferating cells, and its expression increases within the first 24 hours after the induction of terminal differentiation at the time when cells normally exit from the cell cycle. We observed that high levels of Maged1 RNA and protein were maintained as long as the differentiation proceeded. Promoter analysis showed that *Maged1 *transcription was upregulated during myogenic differentiation and that two E-boxes present in the proximal promoter were responsible for this activation. This strongly suggests that MyoD and/or other MRFs is (are) responsible for this regulation.

### Maged1 is required for efficient myoblast cell cycle withdrawal, myogenic differentiation and normal muscle regeneration after injury

The induction of Maged1 during muscle differentiation and regeneration suggested that it could play a role during these processes. We showed that mouse primary myoblasts deficient in Maged1 had a largely reduced capacity to differentiate and to fuse. In vivo, the loss of Maged1 leads to delayed regeneration after muscle injury with the production of smaller muscle fibers. This deficit appears to result from a late block in the differentiation program. Indeed, in the absence of Maged1, we observed normal expression of *MyoD *and *MyoG*, which are early differentiation markers expressed in proliferating cells, whereas the late markers *eMHC *(a muscle structural gene) and *MCK *(an important muscle kinase) were not induced properly. The mechanism responsible for this deficit remains to be fully elucidated, but the reduced ability of the cells to establish the G0 arrest probably contributes to the impaired differentiation. A similar observation was made in P19 embryonic carcinoma cells differentiating in neurons after treatment with retinoic acid: downregulation of Maged1 by morpholinos disturbed neurogenesis by impairing cell cycle withdrawal [22]. Maged1 thus appears to be a key component regulating the transition of progenitors to differentiated neurons and muscle cells by interfering with the cell cycle progression.

A future direction of inquiry following these results will be to determine how Maged1 regulates the G1 to G0 transition in differentiating myoblasts. This cell cycle arrest requires the retinoblastoma tumor suppressor (Rb) protein [23,24]. The precise mechanisms leading to the activation of Rb during myogenesis remain unclear. Rb activity is controlled by its phosphorylation by cyclin-dependent kinases (Cdk), which are in turn controlled by Cdk inhibitors, including p21^CIP1/WAF1 ^[25]. In differentiating myoblasts, p21^CIP1/WAF1 ^transcription is directly controlled by members of the p53 family [26]. Indeed, ectopic expression of ΔNp73, a transdominant inhibitor of all p53 members (p53, p63 and p73) prevents p21^CIP1/WAF1 ^induction, cell cycle withdrawal and myogenic differentiation [26]. The fact that p53 knockout mice exhibit normal muscle development excludes an essential role for this protein in myogenic differentiation and points at p63 and/or p73 as the key factors in this process [26,27]. Additionally, it has been shown that overexpression of Maged1 in HEK293 cells activates p53-dependent transactivation of the *p21^CIP1/WAF1 ^*promoter [28]. As p21^CIP1/WAF1 ^expression is reduced in Maged1-deficient myoblasts, we can hypothesize that Maged1 acts as a transcriptional cofactor of p63 and/or p73 to promote p21^CIP1/WAF1 ^expression and cell cycle exit. Alternatively, Maged1 could act indirectly, by sequestering an inhibitor of p63 and/or p73 activity. Such a mechanism has been shown for Necdin, which sequesters EID-1, an E1A-like protein that inhibits MyoD by suppressing the acetyltransferase activity of its transcriptional co-activator p300 [29].

It has been shown previously that Maged1 can regulate transcription by interacting with several transcription factors and regulators [30-33]. Besides its involvement in the regulation of cell proliferation through p21^CIP1/WAF1 ^expression, Maged1 could directly or indirectly regulate other genes essential for the skeletal myogenic differentiation. A role for Maged1 in cell adhesion has been reported by Xue et al. [34], we could therefore imagine that deficient cell adhesion and/or fusion could also participate in the observed muscle phenotype.

Maged1 is broadly expressed in embryonic and adult tissues and has been identified as interacting with many proteins implicated in apoptosis signaling, cell cycle regulation, cell adhesion and migration, cell differentiation and transcription regulation [12,22,28,31,34-41]. However, the physiological relevance of these interactions remains largely to be demonstrated. We have produced Maged1 knockout mice and have shown previously that Maged1 acts as a pro-apoptotic factor required for normal developmental apoptosis of sympathetic, sensory and motor neurons in vivo [14]. Here, we show that Maged1 plays an important role in myogenic differentiation by allowing myoblasts to express appropriate levels of p21^CIP1/WAF1^, exit the cell cycle and undergo terminal differentiation.

## Methods

### Antibodies and chemicals

The following reagents were purchased as indicated: mouse anti-Nrage (cat. 612500) and mouse anti-BrdU (cat. 555627) from BD Sciences; rabbit anti-MHC from Santa Cruz (cat. Sc-20641); rabbit anti-laminin (cat. L9393) from Sigma-Aldrich; fluorescent secondary Alexa-conjugated antibodies from Invitrogen; horseradish peroxidase-conjugated secondary antibodies from Dako; cell culture media and sera from Lonza Inc.; Dual Luciferase Reporter Assay kit from Promega; Hoeschst 33342 (cat. H1399) and TOPRO^®^-3 (cat. T3605) from Invitrogen; Vectastain ABC kit from Vector Laboratories; other chemicals from Sigma-Aldrich.

### Animals and primary myoblast cell culture

Mice with the conditional floxed allele (*Maged1^tm1Urfm^*) and the Cre-recombined (constitutive knockout) allele for Maged1 have been described previously [14]. *Maged1^tm1Urfm/HSA-Cre ^*mice lacking Maged1 only in differentiating muscle cells were obtained by crossing mice with a floxed Maged1 allele (*Maged1^tm1Urfm/tm1Urfm^*) with transgenic mice specifically expressing Cre recombinase in differentiating myoblasts (Tg(ACTA1-cre)79Jme/J) that were obtained from the Jackson Laboratory [17]. Animals were maintained and treated in compliance with the guidelines specified by the Belgian Ministry of Trade and Agriculture (agreement LA 1900056). Primary myoblasts were isolated from the adult tibialis anterior (TA) muscle of two- to three-month old wild-type or constitutive Maged1 knockout mice following the protocol of the Springer Lab (University of California, San Francisco), which was adapted from the protocol of Springer, M.L., T. Rando, and H.M. Blau (1997) « Gene delivery to muscle » in *Current Protocols in Human Genetics *(John Wiley & Sons, New York). Cells were grown on collagen-coated plates, either in proliferation medium (Ham's F-10 supplemented with 20% FBS, 2.5 ng/ml bFGF, and 50 μg/ml gentamycin) or in differentiation medium (DMEM supplemented with 5% horse serum, 50 μg/ml gentamycin).

### Histology and morphometry

TA muscles of *Maged1^tm1Urfm/HSA-Cre ^*and *Maged1^tm1Urfm ^*adult mice (2-4 months) were injected with 50 μl of 10 μM cardiotoxin (CTX) (from *Naja mossambica mossambica*, C9759 - Sigma-Aldrich) and harvested at the indicated time points. Muscles were fixed in Bouin's solution (for hematoxylin and eosin) or in 4% buffered paraformaldehyde solution (for immunostaining) and were paraffinized following standard protocol. For histological analysis, 6-μm serial sections were stained with hematoxylin and eosin according to standard protocol. To calculate the cross-sectional area of myofibers (XSA), three images were captured from each section and the surface of at least 800 myofibers per genotype per time point were measured using MATLAB (The MathWorks). Laminin immunostaining was performed on 6-μm sections following the instructions of the manufacturer. Nuclei were visualized by counterstaining with Hoeschst 33342.

### Cell lines and plasmids

C2C12 myoblasts were a gift from Professor Raymackers (UCL, Louvain-la-Neuve, Belgium). C2C12 cells were maintained in DMEM supplemented with 20% FBS, 50 μg/ml gentamicin, and a 1x mixture of non-essential amino acids (growth medium, GM). Cells were induced to differentiate in DMEM supplemented with 2% horse serum and 50 μg/ml gentamicin (differentiation medium, DM). NIH-3T3 fibroblasts were cultured in DMEM supplemented with 10% FBS and 50 μg/ml gentamicin.

The MyoD expression plasmid pEMSV-MyoD (original researcher April Hawkins) was a kind gift of Eric N. Olson. Fragments of the human MAGED1 promoter and their mutated versions were amplified by PCR from human DNA and cloned into a pGL3-basic vector (Promega). Site-directed mutagenesis was performed using a triple PCR method. The primers used were the following: -1977 forward: 5'-TCAGAGCTCTATGAAGGAATCGGTTTGGGA-3'; -971 forward: 5'-TCAGAGCTCTTAGTCCCCAGGCCAGCATGA-3'; -531 forward: 5'-TCAGAGCTCTCCTCTCAGGCCATTCCGTT-3'; +80 reverse: 5'-TCAACGCGTGAGGATCGGGATTGCTGGGC-3'; Δ-118 forward: 5'-TCCAGCG*CACA**C**G***A**GCATCATGGTCT-3'; Δ-118 reverse: 5'-AGACCATGATGC**T***C**G**TGTG*CGCTGGA-3'; Δ-227 forward: 5'-GCACAGCG***T**ATC**C**G*T**C**GGGGAGG-3'; Δ-227 reverse: 5'-CCTCCCC**G**A*C**G**GAT**A***CGCTGTGC-3'. Italic sequences indicate E-boxes and letters in bold indicate mutated nucleotides.

### qRT-PCR

Total RNA from cells from 6-cm plates was extracted using the RNeasy mini kit (Qiagen) and subjected to reverse transcription using the Impromt-II transcriptase (Promega) according to the manufacturer's instructions. Quantitative PCR was performed on 7.5 ng of cDNA in a volume of 20 μl with Power SYBR Green PCR Master Mix (Applied Biosystems) using the ABI 7300 real-time PCR System (Applied Biosystems). Unless otherwise cited, primer sequences were designed using Primer Express software (Applied Biosystems).

The primers used were the following: GAPDH forward: 5'-CGTGCCGCCTGGAGAA-3'; GAPDH reverse: 5'-GATGCCTGCTTCACCACCTT-3'; Maged1 forward: 5'-CAAGAGGACCCGCAAGGTT-3'; Maged1 reverse: 5'-GCCTTTGATCCCCACTGTTG-3'; MyoD forward: 5'-GCTGCCTTCTACGCACCTG-3'; MyoD reverse: 5'-GCCGCTGTAATCCATCATGC-3' [42]; MyoG forward: 5'-CCTGGAAGAAAAGGGACTGG-3'; MyoG reverse: 5'-TCATTCACTTTCTTGAGCCTGC-3' [43]; Mef2C forward: 5'-TGGAGAAGCAGAAAGGCACT-3'; Mef2C reverse: 5'-ATTCGTTCCCTCTGCACTTCT-3'; p21 forward: 5'-GGTGGTGGAGACCTGATGAT-3'; p21 reverse: 5'-GCACCTTTTATTCTGCTGGC-3'; eMHC forward: 5'-TGAAGAAGGAGCAGGACAC-CAG-3'; eMHC reverse: 5'-CACTTGGAGTTTATCCACCAGATCC-3' [11]. MCK forward: 5'-GATTCTCACTCGCCTTCGTC-3'; MCK reverse: 5'-GCCCTTTTCCAGCTTCTTCT-3' [44].

### Luciferase Assay

5x10^4 ^NIH-3T3 fibroblasts or C2C12 myoblasts were seeded into each well of 24-well plates and transfected with 1 μg of DNA mixtures, including 20 ng of the *Renilla *luciferase-encoding plasmid pRL-CMV using ExGen500 and Turbofect reagent (Fermentas, Germany), respectively. To determine the activity of MAGED1 during differentiation in C2C12 myoblasts, 980 ng of pMAGED1-1977+80-luc, pMAGED1-971+80-luc or pMAGED1-531+80-luc plasmids were used for each transfection. To analyze the effect of MyoD on the activity of MAGED1 promoter in 3T3 fibroblasts, increasing amounts of the MyoD expression plasmid pEMSV-MyoD were cotransfected with 380 ng of pMAGED1-531+80-luc plasmid. Empty vector (pEMSV) was used as a substitute for pEMSV-MyoD if needed to maintain a constant total amount of transfected DNA. In experiments with mutated MAGED1 promoters in fibroblasts, 600 ng of pEMSV-MyoD or pEMSV were cotransfected with 380 ng of pMAGED1-531+80-luc plasmid or its mutated versions. Cells were harvested at the indicated time points and subjected to luciferase measurement using the Dual Luciferase Reporter Assay kit (Promega). Firefly luciferase activities were normalized to *Renilla *luciferase activities.

### FACS and cell cycle analysis

Cells were harvested by trypsinization, fixed in 70% ethanol overnight at -20°C and incubated with propidium iodide (PI) solution (PI 10 μg/ml, sodium citrate 1.1% and RNAse A 1 mg/ml) for 30 minutes at 37°C before FACS analysis. For each sample, 10,000 cells were analyzed for DNA content in the FL2 channel using FACSCalibur platform (BD Sciences).

### Immunocytochemistry and fusion index

First, 5x10^4 ^primary myoblasts were seeded in each well of 24-well collagen-coated plates in GM. Twenty-four hours later, cells were shifted to DM. To perform immunocytochemistry, cells were fixed in 4% buffered paraformaldehyde solution and incubated with anti-MHC for two hours at room temperature. MHC-expressing cells were visualized using the Vectastain ABC kit (Vector Laboratories). Labeled cells were photographed under a light microscope, and more than 800 nuclei per genotype per time point were counted. The fusion index was calculated by dividing the number of nuclei in MHC-expressing cells containing at least two nuclei by the total number of nuclei of MHC-expressing cells.

### BrdU incorporation test

Cells were incubated for 2 hours with BrdU-containing medium (10 μM) before staining with anti-BrdU and secondary fluorescent antibodies following standard protocol. Nuclei were visualized by counterstaining with TOPRO^®^-3. Approximately 400 cells were counted under fluorescent microscopy to determine the percentage of labeled cells.

### Protein extraction and immunoblot analysis

Cells and muscle tissues were homogenized in RIPA buffer containing 50 mM Tris-HCl pH 7.4, 1% NP-40, 0.25% Na-deoxycholate, 150 mM NaCl, 1 mM EDTA, 1 mM NaF, 1 mM Na_3_VO_4_, and a protease inhibitor cocktail (Complete; Roche Diagnostics) and centrifuged at 1,000 *g *for 10 min at 4°C to discard cellular debris. The protein concentration was measured using the Pierce^® ^BCA Protein Assay Kit. Sample preparation and western blot analysis were performed following standard protocol. Chemiluminescent signals were quantified by digital imaging using the Imagequant 350 system (General Electric)."

### Statistical analysis

All experiments were performed at least three times and results were expressed as mean ± the standard deviation (SD). Statistical analyses were conducted using Student's t-test and the difference was considered significant when the probability value was < 0.05.

## Authors' contributions

T.H.N.N. characterized the expression of Maged1 in myoblasts and regenerating muscles, analyzed the role of Maged1 in myogenesis and muscle regeneration and wrote the manuscript. M.J.M.B. contributed to the production of the Maged1 conditional allele and wrote the manuscript. C.S. helped with the Maged1 promoter analysis. Y.A. helped to obtain transgenic mice. O.R.Y.D.B. designed research, analyzed data and wrote the manuscript. All authors read and approved the final manuscript.

## Supplementary Material

Additional file 1 **Figure S1. Cell-cycle distribution of C2C12 myoblasts 12 h and 24 h after serum starvation**. C2C12 myoblasts were induced to differentiate by serum starvation, and the fractions of cells in G1, S and G2/M were determined by FACS after staining with propidium iodide. Click here for file

Additional file 2 **Figure S2. MyoD activates the *MAGED1 *promoter in a dose-dependent manner**. Increasing amounts of MyoD expression plasmid pEMSV-MyoD were cotransfected with pMAGED1-531+80-luc in 3T3 fibroblasts. Luc activity was measured 48 h after transfection. Results are presented as relative Luc activities with respect to the activity of pMAGED1-531+80-luc in absence of MyoD. Click here for file

Additional file 3 **Figure S3. Maged1 knockout myoblasts express normal levels of MyoD, MyoG and Mef2C**. Wild-type and Maged1 knockout primary myoblasts were induced to differentiate by serum starvation. *MyoD, MyoG and Mef2C *RNA levels were quantified using qRT-PCR. *GAPDH *RNA was used for normalization. Data are presented as the ratio relative to the RNA levels of each analyzed gene in wild-type cells at the time of serum starvation (t = 0 h). Click here for file
