# Supplementary figures and images for: Maged1, a new regulator of skeletal myogenic differentiation and muscle regeneration
Source: BMC Cell Biol. 2010 Jul 20;11:57. doi: 10.1186/1471-2121-11-57 (PMC2912822; doi:10.1186/1471-2121-11-57)

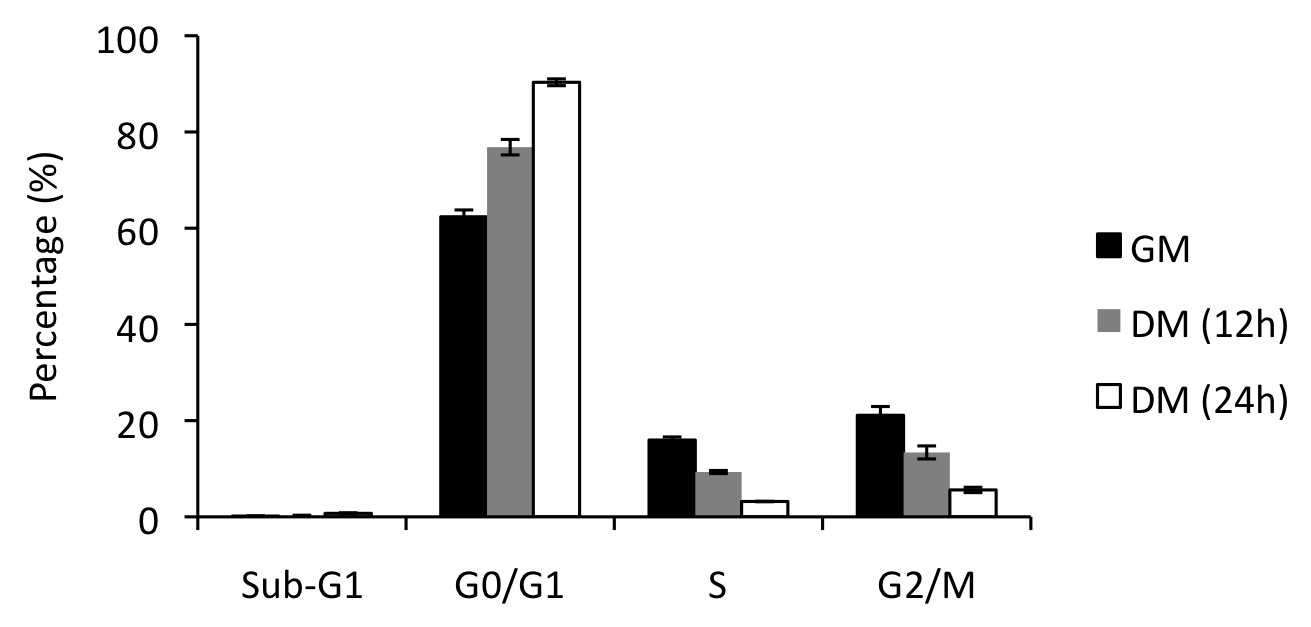

Supplement: Additional file 1 — Figure S1. Cell-cycle distribution of C2C12 myoblasts 12 h and 24 h after serum starvation. C2C12 myoblasts were induced to differentiate by serum starvation, and the fractions of cells in G1, S and G2/M were determined by FACS after staining with propidium iodide. [file 1471-2121-11-57-S1.JPEG]

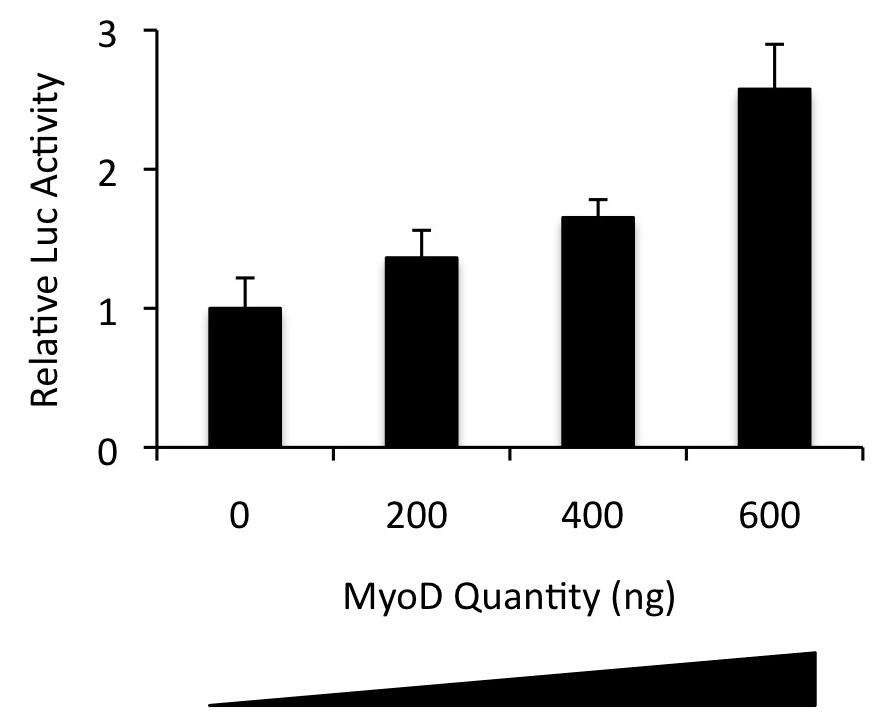

Supplement: Additional file 2 — Figure S2. MyoD activates the MAGED1 promoter in a dose-dependent manner. Increasing amounts of MyoD expression plasmid pEMSV-MyoD were cotransfected with pMAGED1-531+80-luc in 3T3 fibroblasts. Luc activity was measured 48 h after transfection. Results are presented as relative Luc activities with respect to the activity of pMAGED1-531+80-luc in absence of MyoD. [file 1471-2121-11-57-S2.JPEG]

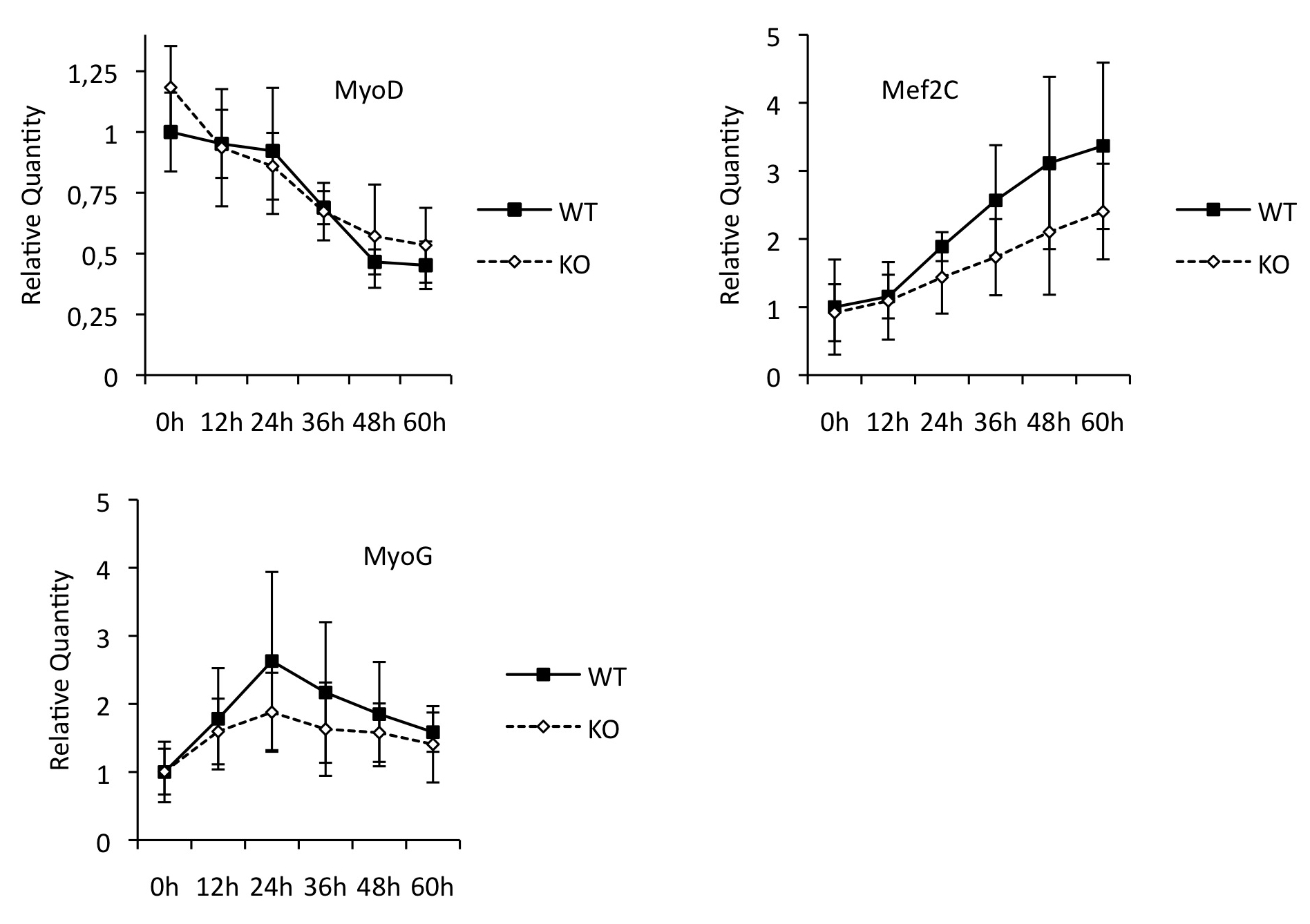

Supplement: Additional file 3 — Figure S3. Maged1 knockout myoblasts express normal levels of MyoD, MyoG and Mef2C. Wild-type and Maged1 knockout primary myoblasts were induced to differentiate by serum starvation. MyoD, MyoG and Mef2C RNA levels were quantified using qRT-PCR. GAPDH RNA was used for normalization. Data are presented as the ratio relative to the RNA levels of each analyzed gene in wild-type cells at the time of serum starvation (t = 0 h). [file 1471-2121-11-57-S3.JPEG]
